# Ketamine as part of multi-modal analgesia may reduce opioid requirements following cardiac surgery: a retrospective observational cohort study

**DOI:** 10.1186/s13019-025-03405-x

**Published:** 2025-04-03

**Authors:** James Hall, Juri Chung, Michael Khilkin, George Elkomos-Botros

**Affiliations:** 1 Cardiothoracic Critical Care, Department of Cardiac Surgery, NYU Langone, Long Island Hospital, 259 1st Street, Mineola, New York, 11501 USA; 2 Critical Care Pharmacy, NYU Langone, Long Island Hospital, 259 1st Street, Mineola, New York, 11501 USA

**Keywords:** Ketamine, Multimodal analgesia, Opioid-sparing effect, Postoperative pain management

## Abstract

**Background:**

Postoperative pain control in cardiac surgery is often managed with opioid medications. Insufficient analgesia can result in complications including splinting, pneumonia, and delay of appropriate rehabilitation. Given the risks and adverse effects of opioids including sedation, respiratory depression, delirium, and decreased gastrointestinal motility, hyperalgesia and potential for addiction, strategies for opioid reduction are likely to improve outcomes, therefore multimodal opioid sparing pain regimens are preferred. Recently, there is increased evidence that low dose Ketamine, an N-methyl-D-Aspartate (NMDA) receptor antagonist, is safe and effective for analgesia in postoperative patients and may be appropriate to this setting.

**Methods:**

This is a single center, retrospective, observational, cohort study over a one year period involving postoperative adult cardiac surgery comparing those who received a single dose of postoperative ketamine, 0.3 mg/kg over 30 min, with those who did not receive any ketamine. Other analgesic protocols were similar between groups and did not include additional ketamine. A total of 120 patient charts were reviewed, of which 96 met inclusion criteria, 32 in the ketamine group and 64 in the standard care group. Charts were reviewed for opioid and other pain medication requirements during the postoperative period and on discharge, and for secondary outcomes: hospital length of stay, ICU length of stay, in-hospital and 30-day mortality, 30-day readmission, and rates of delirium, emergence reactions, and need for escalated respiratory support.

**Results:**

The group who received postoperative ketamine required 28.8 morphine milligram equivalents (MME) less postoperative opioid (90.1 mg vs 118.9 mg, *p* = 0.167), and was prescribed an average of 15.8 MME less on discharge (*p* < 0.001). Intraoperatively, both groups received 1000 mg acetaminophen, 2 mg intravenous morphine and 100 mcg fentanyl, 26 MME, by protocol. No ketamine was administered intraoperatively or preoperatively. The groups differed in respect to operation type and controlling for this parameter failed to achieve significance in needs during admission (*p* = 0.215), but remained significant on discharge (*p* = 0.02). The non-ketamine group received more ketorolac (15.5 vs 10.1, *p* = 0.06). The ketamine group required less acetaminophen but more gabapentin. There was no difference in hospital or ICU length of stay. There was no delirium or mortality in either group. Respiratory depression occurred in 15 patients who all subsequently received ketamine. No patient developed respiratory depression after ketamine.

**Conclusions:**

Ketamine may be a reasonable choice for postoperative cardiac surgery analgesia and may reduce the need for opioids on discharge, and possibly during admission.

## Background

Postoperative pain after cardiac surgery, involving the highly innervated thoracic wall, can be severe and failure and contribute to significant postoperative complications including splinting, pneumonia, delirium of critical illness, failure to mobilize or rehabilitate appropriately, and a prolonged hospital stay [1]. While traditionally opioid medications have been the cornerstone of postoperative pain control, they entail significant risk for adverse effects including sedation, respiratory depression, delirium, and decrease gastrointestinal motility, opioid induced hyperalgesia, and the potential for addiction [2, 3]. Consequently, a focus on combinations of non-opioid adjunct analgesics–termed multimodal analgesia–intended to decrease opioid requirements has emerged as the standard for post cardiac surgery analgesia. One potentially useful agent is ketamine. Alone or in combination with other analgesics, it has been used to treat moderate to severe pain in a variety of settings, including postoperatively. Relevant guidelines including those published by the Enhanced Recovery after Surgery (ERAS) program recommend an opioid sparing multimodal analgesia approach which includes acetaminophen, gabapentin, and dexmedetomidine. The latest ERAS guidelines in 2019 identified ketamine as having potential for inclusion, but citing limited evidence, suggested further study [4].

First described in 1965 and approved by the FDA in 1970, ketamine (derived from ‘ketone-amine’) is an N-methyl, D-aspartate (NMDA) receptor antagonist chemically related to phencyclidine that exerts its effect by modulating the central nervous system’s (CNS) sensory experience of pain by binding to multiple sites including the µ and δ opioid receptors [5]. It is classified as a dissociative anesthetic.

Ketamine has been found to have several dose-dependent therapeutic effects. At low doses (0.1–0.5 mg/kg slow bolus or up to 1 mg/kg/hr continuous infusion), it has been shown to be effective for the treatment of moderate to severe pain without significant sedative or hemodynamic effects. At moderate doses, it retains its analgesic effects but also produces dose dependent amnesia and dissociative sedation, again without significant hemodynamic effects. At higher doses, it is used for anesthetic induction (1–2 mg/kg bolus), general anesthesia, and in the treatment of refractory status epilepticus (2–3 mg/kg/hr) [6]. At these doses, it has been noted to be a cardiovascular stimulant, resulting in increased blood pressure and heart rate. While the side-effect profile is generally favorable, at the moderate and higher doses, it has additionally been found to cause variable psychosensory aberrations and a sense of dissociation as well as and temporary memory problems, and can increase airway secretions. Previous concerns about increases in intracranial pressure have been shown to result from increases in cerebral blood flow, augmenting perfusion, rather than from extravascular edema [5].

As an analgesic, low dose ketamine has been shown to be an effective in the setting of opioid induced hyperalgesia. This complication is thought to occur as a result of NMDA receptor activation by natural and synthetic opioids resulting in opioid tolerance and increased perception of pain. This mechanism putatively suggests ketamine’s observed utility in prophylaxis and treatment of this problem [7]. Ketamine administration has also been shown to be associated with lower levels of pro-inflammatory cytokines and higher levels of anti-inflammatory cytokines. This association is postulated to be responsible for ketamine’s observed protective effect against ICU delirium [8]. The occurrence of side effects from ketamine has been shown to be dependent on both the dose and the rate of administration. However, side effects seem to be limited to the duration of administration and disappear shortly after discontinuation. In contrast, the analgesic and anti-depressive benefits may have a prolonged effect [9, 10]. As such it has been used in the treatment of both acute and chronic pain, and has now been approved by the FDA in the treatment of depression [11].

As a postoperative analgesic, the reported results on ketamine are mixed. Some studies have shown a significant reduction in acute and chronic postoperative pain and a reduction in opioid pain medication requirements [12, 13]. Others have shown little to no benefit [14]. Relatively few studies have been conducted in the cardiac surgery population, but in the published literature, study protocols have varied considerably with respect to timing, dose, and setting (intraoperative vs postoperative), but have thus far failed to demonstrate a decreased need for opioids. A recent randomized control trial conducted in children undergoing open heart surgery did show significantly less opioid use with ketamine use [15]. It has also been shown that ketamine decreases the inflammatory response associated with cardiopulmonary bypass and reduces the subsequent associated cognitive dysfunction [8, 16]. A Cochrane Database systematic review examining postoperative ketamine concluded that it, “probably reduces postoperative analgesic consumption and pain intensity,” and that there was no significant difference in CNS adverse events [17].

Because the existing data in cardiac surgery are conflicted regarding the degree of benefit from postoperative ketamine in the management of postoperative pain, and because optimal timing, dose, duration, and route are unclear, further examination is warranted. This study specifically examines the effect of the administration of a single dose of postoperative ketamine on the subsequent need for opioid medications and other analgesics after cardiac surgery both in-hospital and upon discharge.

## Methods

We performed a retrospective, observational, chart review study of adult patients admitted for elective cardiac surgery at NYU Langone Hospital – Long Island from January to December, 2022. These dates were chosen based on changes in the analgesic protocols at the institution. Prior to this period, little ketamine was used; following this period, ketamine was more often administered as a continuous infusion. The purpose of this investigation was to evaluate whether administration of 0.3 mg/kg of ketamine over 30 min in the immediate postoperative period in addition to standard multimodal analgesia (acetaminophen, gabapentin, and methocarbamol) reduced overall postoperative opioid requirements as measured in MME from the time of surgery to discharge. We also examined what association this dose may have had with post-discharge opioid requirement, what effect it may have had on the administration of non-opioid analgesics, and whether it was associated with ICU and hospital length of stay, mortality, readmission, respiratory support escalations or delirium. The target population was patients undergoing uncomplicated cardiac surgery. Adult patients were included if they underwent a scheduled cardiac procedure, were admitted to the cardiac surgery intensive care unit, and were not severely ill postoperatively, as defined by prolonged (> 24 h) mechanical ventilation, prolonged vasopressor or inotrope requirements, mechanical circulatory support, and reoperation or mortality within 24 h of surgery. Cardiac procedures included coronary artery bypass graft (CABG), aortic or mitral cardiac valve surgery (AVR, MVR), and ascending aortic surgery or a combination of these. By clinical protocol, all patients received 1000 mg IV acetaminophen, 100 mcg of fentanyl and 2 mg morphine intraoperatively as well as an intercostal nerve block. Operating room anesthetics were sevoflurane and propofol. No patient received additional intraoperative analgesia including ketamine.

We separated these patient into groups based on whether they received ketamine postoperatively. The decision to administer ketamine was at the discretion of the treating team, and the indication was for postoperative pain; no doses were administered prophylactically, and no ketamine was administered for sedative purposes. Ketamine doses were administered at 0.3 mg per kilogram as an intravenous infusion over 30 min. As this study was retrospective, there was no blinding or randomization. All patients who received ketamine were awake and extubated. Intraoperative MME was not included in the study. We verified that no patient received ketamine before or during their operations. In addition to whether they received ketamine, all postoperative patients were managed with multimodal analgesia including acetaminophen, methocarbamol, gabapentin and opioid analgesics as needed.

Chart review procedure involved obtaining a list of all cardiac surgery patients at NYU Langone, Long Island Hospital during the study timeframe (Jan-Dec, 2022) and screening them by the inclusion and exclusion criteria. Manual chart review was then conducted for demographic variables which included age, sex, race, weight, past medical history, social history, use of opioids or on treatment for opioid dependence. Opioid doses were recorded and converted to MME for standardization. Doses of non-opioid analgesics were also recorded. Outcome measures included cumulative opioid dose in MME and cumulative non-opioid medications requirements from the postoperative admission to the ICU through hospital discharge. Secondary outcome measures included the occurrence of delirium or emergence reactions, respiratory decompensations as determined by the need for an escalation in mode of respiratory support, any in-hospital or 30-day mortality or 30-day re-admission.

Group characteristics were compared with cross-tabulation and chi squared analysis. Outcome measures were compared between the groups with cross-tabulation and chi squared for categorical variables or independent t-test or regression for continuous variables as appropriate. As baseline characteristics were found to differ significantly for procedure type, additional analyses with analysis of variance (ANOVA) and linear regression models were run controlling for this potential confounder. Values were reported as means and medians with 95% confidence intervals. Significance level was set to a p-value of < 0.05. All analyses were performed using SPSS Statistics version 28 software (IBM, Armonk, NY).

## Results

A total of 120 patient charts were identified as meeting inclusion criteria, of which 24 were subsequently excluded for significant instability, prolonged mechanical ventilation or incomplete or missing data. Ninety-six patient charts were reviewed. Thirty-two patients received a dose of ketamine and 64 did not. Baseline characteristics were largely similar and are given in Table 1. There was, however, a statistically significant difference in the procedure type *p* < 0.001. The ketamine group including 43.7% with CABG, 37.5% with valve replacement or repair while the non-ketamine group was almost exclusively CABG patients (95.3%). The ketamine group also included more black patients (12.5% vs 1.6%, *p* = 0.023). There were non-statistically significant differences in age, sex, and history with the ketamine group tending to be slightly younger (64.8 years old vs 67.5, *p* = 0.054), more female (28.2% vs 15.6%, *p* = 0.147), with more prior malignancy (15.6% vs 6.3%, *p* = 0.137), and less prior smoking (12.5% vs 25%, *p* = 0.155). Active smokers were similar between groups (9.4% vs 12.5% *p* = 0.65). All other variables were well-matched between the two groups. As a result of the differences, additional statistical analyses were run to control for procedure type. Miscellaneous procedures in the ketamine group included one combined CABG/MVR, one mitral valve repair, two pericardial drain placements, one reimplantation of an anomalous right coronary artery, and one thoracotomy for removal of a displaced appendage closure device, and other procedures in the non-ketamine group included one combined CABG/AVR and one combined CABG/MVR.

**Table 1:** Baseline characteristics of both those who did and did not receive ketamine

	Ketamine plus standard analgesia	Standard analgesia	*p*-value
Mean Age (range)	64.8 (46–78)	67.5 (44–85)	0.054
Male (%)	23 (71.8)	54 (84.4)	0.147
*Ethnicity*			
White (%)	24 (75)	52 (81.3)	0.477
Asian (%)	2 (6.3)	7 (10.9)	0.458
Hispanic (%)	2 (6.3)	2 (3.1)	0.47
Black (%)	4 (12.5)	1 (1.6)	0.023
Unknown (%)	0	2 (3.1)	0.542
*Procedure*			
CABG (%)	14 (43.7)	61 (95.3)	< 0.001
Valve (%)	12 (37.5)	1 (1.6)	< 0.001
Other (%)	6 (18.8)	2 (3.1)	0.01
*Past medical history*			
Coronary disease (%)	13 (40.6)	24 (37.5)	0.767
Hypertension (%)	22 (68.8)	50 (78.1)	0.317
Hyperlipidemia (%)	19 (59.4)	49 (76.6)	0.081
Diabetes (%)	8 (25)	24 (37.5)	0.221
COPD (%)	1 (3.1)	5 (7.8)	0.371
Asthma (%)	2 (6.3)	3 (4.7)	0.745
Chronic renal disease (%)	1 (3.1)	7 (10.9)	0.192
Atrial fibrillation (%)	4 (12.5)	4 (6.3)	0.296
Rheumatic disease (%)	1 (3.1)	0	0.155
Prior malignancy (%)	5 (15.6)	4 (6.3)	0.137
Immunosuppressives (%)	2 (6.3)	1 (1.6)	0.213
*Social history*			
IV drug abuse	0	0	NA
Alcohol (%)	0	2 (3.1)	0.312
Current smoker (%)	3 (9.4)	8 (12.5)	0.65
Former smoker (%)	4 (12.5)	16 (25)	0.155

In this patient cohort, the majority of patients who received ketamine did so within a few hours of the operation. Twenty-four patients (75.0%) were administered it on postoperative day zero, 6 patients (18.8%) on day 1, and 2 patients (6.3%) on day 2. All patients who received ketamine were awake and extubated at the time and the decision to use pain-dose ketamine was solely at the discretion of the treating team.

**Fig. 1:**
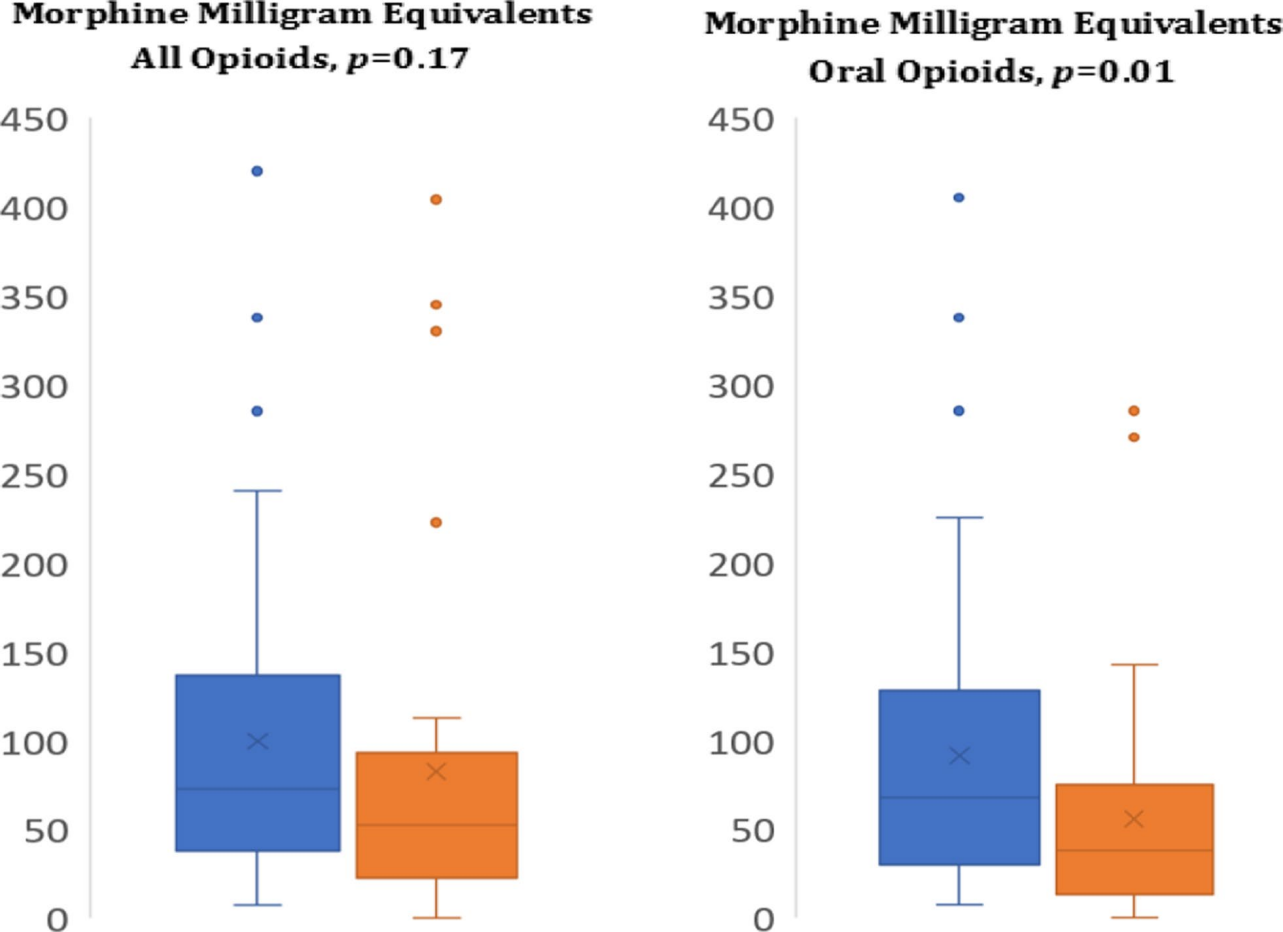
Distribution of inpatient and outpatient opiate requirements between groups

All patients who received ketamine had access to narcotics as needed to achieve adequate pain control. As expected, the mean post-treatment pain scores on postoperative day (POD) 1 did not differ between groups (ketamine 1.4 SD 1.2 vs non-ketamine 1.6 SD 1.1, *p* = 0.63). The primary outcome, total postoperative opioid administration in morphine milligram equivalent (MME) was less by 28.8 units (90.1 vs 118.9) in those who received ketamine vs those who did not (*p* = 0.167, − 30.0 to 87.6 95% CI), though this failed to achieve statistical significance (Fig. 1). When controlled for procedure type, this difference remained non-significant (*p* = 0.215). Additional analysis controlling for administered ketorolac dose, which was higher in the non-ketamine group (15.5 mg vs 10.1 mg), the effect was more pronounced, but still failed to achieve significance (*p* = 0.07).

The in-hospital and 30-day mortality in both groups was zero. Median ICU length of stay (2 days vs 2 days) and median postoperative hospital length of stay (6 days vs 6 days) were the same in both groups. Thirty day readmission rates were similar (3 (9.4%) vs 1 (1.6%) *p* = 0.071). No patients in either group developed delirium or emergence reaction and none was treated for agitation. Of note, 15 patients of the 96 required escalation of their respiratory support (nasal cannula to high flow nasal cannula to BiPAP) and all were in the ketamine group, but all of these escalations occurred prior to the administration of ketamine. We speculate that opioid avoidance related to further respiratory deterioration may have been a trigger for ketamine use in these patients. No patient required reintubation (Tables 2 and 3).

**Table 2:** Concomitant medication requirements in multimodal analgesia

Concomitant medications	Ketamine	Standard	Difference (95% CI)	*p*-value
Acetaminophen daily dose	3406 mg	3787 mg	− 381.1 (− 712.0 to − 50.1)	0.012
Gabapentin daily dose	559.4 mg	190.6 mg	368.8 (201.0 to 536.5)	< 0.001
Methocarbamol daily dose	1367.2 mg	1093.8 mg	273.4 (− 137.0 to 683.9)	0.09
Ketorolac total dose	10.1 mg	15.5 mg	− 5.4 (− 16.2 to 5.4)	0.06
Oxycodone total dose	20.7 mg	48.7 mg	− 28.0 (− 48.9 to − 7.0)	0.01
Hydromorphone total dose	0.14 mg	0.07 mg	0.07 (− 0.04 to 0.18)	0.11
Fentanyl total dose	21.5 mcg	17.8 mcg	3.7 (− 15.6 to 23.0)	0.35
Methadone	0	0	NA	NA

**Table 3:** Primary and secondary outcomes between groups

	Ketamine	Standard	Difference (95% CI)	*p*-value
*Primary outcome*				
Inpatient opioid requirements (MME)	90.1	118.9	− 28.8 (− 87.6 to 30.0)	0.167 Controlled: 0.215
Outpatient opioid requirements (MME)	15.7	31.4	− 15.8 (− 25.6 to − 5.9)	0.001 Controlled: 0.02
*CABG Only subgroup*				
Inpatient opioid requirements (MME)	76.7	124.4	− 47.7 (− 129.1 to 33.7)	0.123
Outpatient opioid requirements (MME)	20.5	29.7	− 9.21 (− 23.3 to 4.90)	0.029
*Secondary outcomes*				
Hospital LOS mean (median)	6.7 (6)	7.0 (6)		0.623
ICU LOS mean (median)	2.8 (2)	2.4 (2)		0.045
In-hospital Mortality	0	0		–
30-day mortality	0	0		–
30-day readmission (%)	3 (9.4)	1 (1.6)		0.071
Delirium or emergence	0	0		–
Requiring additional respiratory support?	15 (15.6)			< 0.001

All patients received the non-opioid multimodal analgesia as per existing protocol: acetaminophen, gabapentin, and methocarbamol. Eighteen patients (56.2%) in the ketamine group and 29 patients (45.3) in the non-ketamine group received ketorolac. A few patients in both groups received ketorolac at the discretion of the treating team. There was a statistically significant difference in the dose of acetaminophen administered. The ketamine group received 381.1 mg less than the non-ketamine group (50.1–712.0 95% CI, *p* = 0.012). However, the ketamine group was given 368.8 mg more gabapentin (201.1–536.5 95% CI, *p* < 0.001). Those in the ketamine group received less ketorolac than those in the non-ketamine group (15.5 mg (0–135 mg) vs 10.1 mg (0–20 mg) *p* = 0.06). Oxycodone administration was significantly different between the groups with the ketamine group receiving 57.5% less (28.0 mg) than the non-ketamine group (20.7 mg vs 48.7 mg, *p* = 0.01) (Tables 2 and 3).

**Fig. 2:**
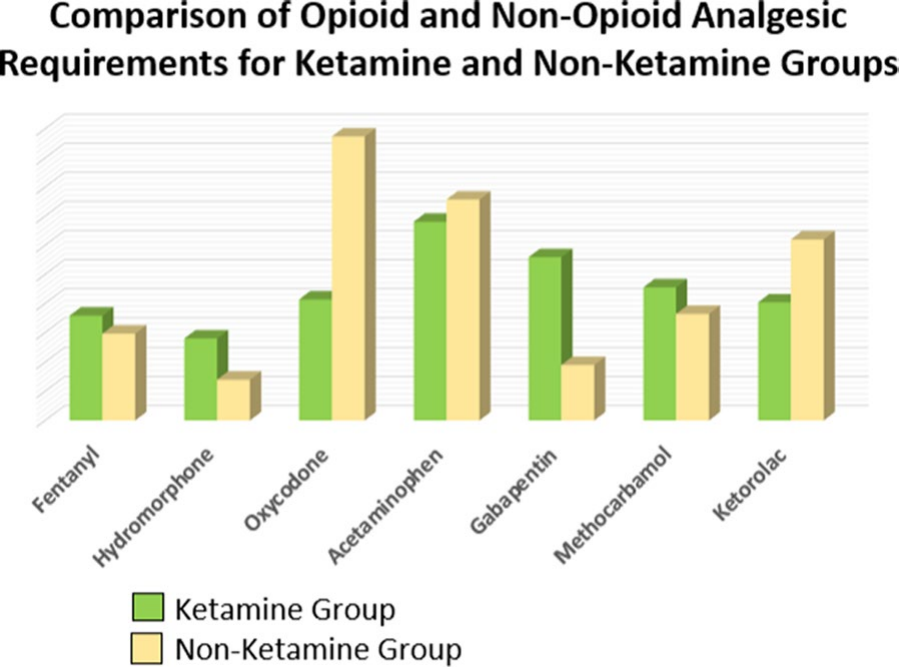
Relative doses of analgesics between groups

A similar proportion of patients required opioid prescriptions on discharge, 59% of patients in the ketamine group and 68.8% in the non-ketamine (*p* = 0.471), but the patients in the ketamine group received a significantly lower quantity on discharge, 15.7 MME vs 31.4 MME, a difference of 15.8 MME (5.9–25.6 95% CI, *p* = 0.001). When controlling for ketorolac dosing, discharge dose difference was slightly less, but remained significant (*p* = *0.*004) (Fig. 2). When controlled for procedure type, this effect was also significant (*p* = 0.02).

## Discussion

The primary outcome for this study was overall opioid requirements in the post-operative period. The findings failed to show a statistically significant difference between total opioid requirements in the in-patient postoperative course after ketamine administration, but did demonstrate a significant decrease in the oral opioid (oxycodone) administration. When including intravenous opiates, the results trend toward a reduction in opioids, but the study is underpowered to demonstrate significance. There were no differences in pre-operative or intraoperative management. The ketamine group received more gabapentin, but less acetaminophen and ketorolac. The reasons for the providing teams’ preference for one over the other in these groups is unclear. As noted above, it appears that escalating respiratory requirements may have steered teams away from choosing opioids and towards using ketamine, and that this choice did not result in any worsening in the respiratory status. On discharge, the ketamine group was prescribed less oxycodone. Discharge prescriptions at our institution are based on analgesic requirements in the 48 h preceding discharge suggesting that the ketamine doses in the early postoperative course may have had downstream effects. The influence of ketorolac, a pronounced non-steroidal anti-inflammatory medication, used more in the non-ketamine group was apparently insufficient to offset the differences from ketamine. This may be due to the attenuation of the opioid induced hyperalgesic response by NMDA blockade. This could potentially lead to less subsequent pain and lower requirements [7]. Ketamine is also known to cause a relative shift in the cytokine balance if favor of less inflammation [8]. However, ketorolac, a pronounced non-steroidal anti-inflammatory medication, used more in the non-ketamine group which still had higher oral opiate requirements. The unique effects of ketamine are still being studied and further research may elucidate further downstream mechanisms contributing to this effect.

Despite the primary outcome not reaching significance, the findings of this study may provide some support to the hypothesis that ketamine may reduce the need for opioid pain medication in cardiac surgery patients both inpatient and on discharge. If further validated, ketamine may be reasonably included in an opioid sparing multimodal analgesic approach in this population.

Our findings are partially corroborated by existing literature. Aguirreche, et al. demonstrated lower postoperative opioid requirements in cardiac surgery patients when a multimodal analgesic approach including ketamine was used intraoperatively [18]. Lahtinen, et al. observed a decreased opioid requirement in those receiving a bolus and low dose infusion of ketamine after cardiac surgery [19]. Anwar, et al. reported that both gabapentin and to a larger extent pregabalin plus ketamine perioperatively significantly lowered cardiac surgery patient’s pain scores at 3 and 6 months [20]. However, other studies did not demonstrate significant benefit. One found no difference in opioid use with an intraoperative ketamine bolus and drip, and another found no difference in pain scores at 9 months with a very low intraoperative drip [14, 21].

This stands in contrast to studies done in other surgical patient populations where results have been generally favorable to the use of ketamine. Better pain control and opioid sparing effects have been noted in patients undergoing mastectomy, abdominoplasty, gastric bypass, orthopedic, and spine surgery [12, 13, 22–24].

The hypothesis that a pre-incisional dose of ketamine may be helpful to attenuate later pain has also been tested. Safavi, et al. demonstrated a reduction in opioid requirements with a pre-incisional dose of ketamine administered as either an intravenous bolus or a subcutaneous infiltration at the site of incision, but Cameron, et al. found no difference when low dose pre-incisional and intraoperative ketamine infusions were administered [21, 25]. Most recently, a 2024, study done in children undergoing open heart surgery reported that a low dose ketamine infusion beginning before incision and continued for 48 h postoperatively demonstrated a significant reduction in post-operative opioid requirements [15].

Beyond purely surgical studies, a significant improvement in a variety of chronic pain syndromes with intermittent ketamine administration has been demonstrated [9]. While the pathways involved in chronic pain are complex, such findings may suggest some durable benefit to ketamine use. A 2019 meta-analysis of studies evaluating the use of ketamine for chronic pain demonstrated a small but persistent effect up to two weeks following administration [26]. A meta-analysis from 2023 found similar results and that ketamine at pain doses (0.1–0.49 mg/kg) were effective at controlling pain. Additionally, the same meta-analysis examined the incidence of sedation, nausea, and neuropsychiatric side-effects, commonly cited concerns in the use of ketamine, and found no significant difference from control groups [27]. A meta-analysis in non-cardiac surgical patients found up to 50% reduction in opioid use when ketamine was used as part of a multimodal analgesic strategy [9].

In 2018, a Cochrane meta-analysis that included 130 studies and 8,341 participants, but no cardiac surgery patients, reported improved pain and a reduction in nausea without any significant central nervous system differences [17]. Ketamine, in fact, has shown improved cognitive function and reduced delirium, potentially mediated by observed reductions in proinflammatory cytokines and increases in anti-inflammatory cytokines [8, 16, 28]. However, one small study reported significant rates of hallucinations and one larger trial showed no increase in delirium, but an increase in unpleasant dreams [19, 29]. As the literature remains conflicted, our study of a single administration in the postoperative period helps to bridge the gap between intraoperative data and postoperative infusion data.

A particularly promising use of ketamine is in patients with previous opiate use or dependence. The physiologic tolerance and diminishing analgesic returns of long term opioid use are well described. In addition to preventing opioid induced hyperalgesia, NMDA receptor blockade has been credited in resensitizing patients to opioids and resetting the nociceptive baseline. NMDA antagonists, such as ketamine, have been found to be effective analgesics both in patients with long term opioid desensitization and opioid hyperalgesia syndrome [3, 30].

It is also important to address that certain unique barriers to low-dose ketamine exist within systems. In addition to the unfamiliarity and resistance to change that comes with any innovation, ketamine has historical and social hurdles to overcome. Ketamine is classified as a dissociative anesthetic, and many institution have specific policies about settings and providers entitled to order anesthetics. This has led to a lack of familiarity with it among many providers and a resultant discomfort. Moreover, ketamine also suffers from negative stereotypes due to its potential for abuse as a street drug, its known hallucinatory properties at higher doses. Because ketamine has a relatively favorable safety profile, it has often been used in non-hospital settings such as pediatric clinics, dental offices and in veterinary medicine where it earned a reputation as a “horse tranquilizer” [31]. In spite of these challenges, expert opinion is favorable. The 2018 joint consensus statement from the American Society of Regional Anesthesia and Pain Medicine, the American Academy of Pain Medicine, and the American Society of Anesthesiologists recommend that ketamine be considered for perioperative surgical pain as a stand-alone or adjunct agent [32].

While we consider our findings to be in support of this view, there are a number of significant limitations to the study. Methodologically, as a retrospective observational study, there were no bias controls such as randomization, blinding, or placebo counterfactuals. Selection bias, as is implied by its disproportional use in patients with increased respiratory support prevent us from identifying patients who might preferentially benefit. Further variability was introduced related to various providers’ familiarity and comfort level with ketamine. Staff protocols, long dependent on opioids for pain control may have administered more based on expectations rather than actual needs. All of these would act to dampen clinical effect size and create more noise in the data, and potentially underestimate the actual benefits of ketamine. There is significant need for additional studies to clarify dosing, timing, duration, and whether concomitant pre-incisional and intraoperative dosing might lead to greater efficacy. Optimally, these would be prospective studies with appropriate controls to reduce bias.

Broadly, these findings and relevant concurring literature suggest that ketamine may be a reasonable option as part of multimodal analgesia in cardiac surgery and may be a useful in limiting opioid requirements in the post-operative and post-discharge phases of care. Optimizing multimodal analgesia is an important area for ensuring the best possible outcomes. We agree with several recent publications that with appropriate adjustments in agents, dosing and timing, it may be possible to significantly reduce and even eliminate the need for opioids with their problematic cadre of potential complications in this high risk population [33, 34].

## Conclusion

Low dose ketamine for analgesia may be a reasonable choice in uncomplicated postoperative cardiac surgery patients in order to facilitate adequate pain control and reduce the need for opioid pain medications in both the post-operative in-patient phase of care and post discharge.

## Data Availability

The datasets used and/or analyzed during the current study are available from the corresponding author on reasonable request.
